# Primary pancreatic paraganglioma: a case report and literature review

**DOI:** 10.1186/s12957-016-0771-2

**Published:** 2016-01-22

**Authors:** Shengrong Lin, Long Peng, Song Huang, Yong Li, Weidong Xiao

**Affiliations:** 1 Department of General Surgery, The First Affiliated Hospital of Nanchang University, No.17 Yongwai Zhengjie, Nanchang, 330006 Jiangxi China; 2 Department of Pathology, The First Affiliated Hospital of Nanchang University, No.17 Yongwai Zhengjie, Nanchang, 330006 Jiangxi China

**Keywords:** Pancreas, Paraganglioma, Middle segment pancreatectomy

## Abstract

**Backgroud:**

Primary pancreatic paraganglioma is an extremely rare extra-adrenal paraganglioma.

**Case presentation:**

We report a case of primary pancreatic paraganglioma undergoing middle segment pancreatectomy in a 42-year-old woman. Histological examination showed that the tumor was composed of well-defined nests of cuboidal cells separated by vascular fibrous septa, forming the classic Zellballen pattern. The chief cells showed positive staining to neuron-specific enolase, chromogranin A, synaptophysin, and the chief cells were surrounded by S-100 protein-positive sustentacular cells. The patient has remained tumor free for 12 months after surgery. A brief discussion about the histopathological features, clinical behavior, and treatment of primary pancreatic paraganglioma, and review of the relevant literature is presented.

**Conclusions:**

Primary pancreatic paraganglioma is a rare clinical entity, its diagnosis mainly depends on histopathological and immunohistochemical examinations. Complete surgical resection is the first choice of treatment and close postoperative follow-up is necessnary.

## Background

Paragangliomas are rare neuroendocrine tumors (NETs) that arise from the extra-adrenal chromaffin cells of the autonomic nervous system, with an average annual incidence rate of only 2 to 8 per 1 million adults. Paragangliomas could derive from the extra-adrenal chromaffin cells of the sympathetic paravertebral ganglia of the thorax, abdomen, and pelvis, also arise from the parasympathetic ganglia located along the glossopharyngeal and vagal nerves in the neck and at the base of the skull. However, primary pancreatic paraganglioma is extremely rare. Herein, we present a case of primary pancreatic paraganglioma and review of the literature.

## Case presentation

**Fig. 1:**
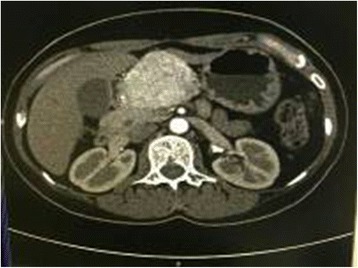
On contrast-enhanced CT, the tumor demonstrated marked enhancement in the arterial phase

**Fig. 2:**
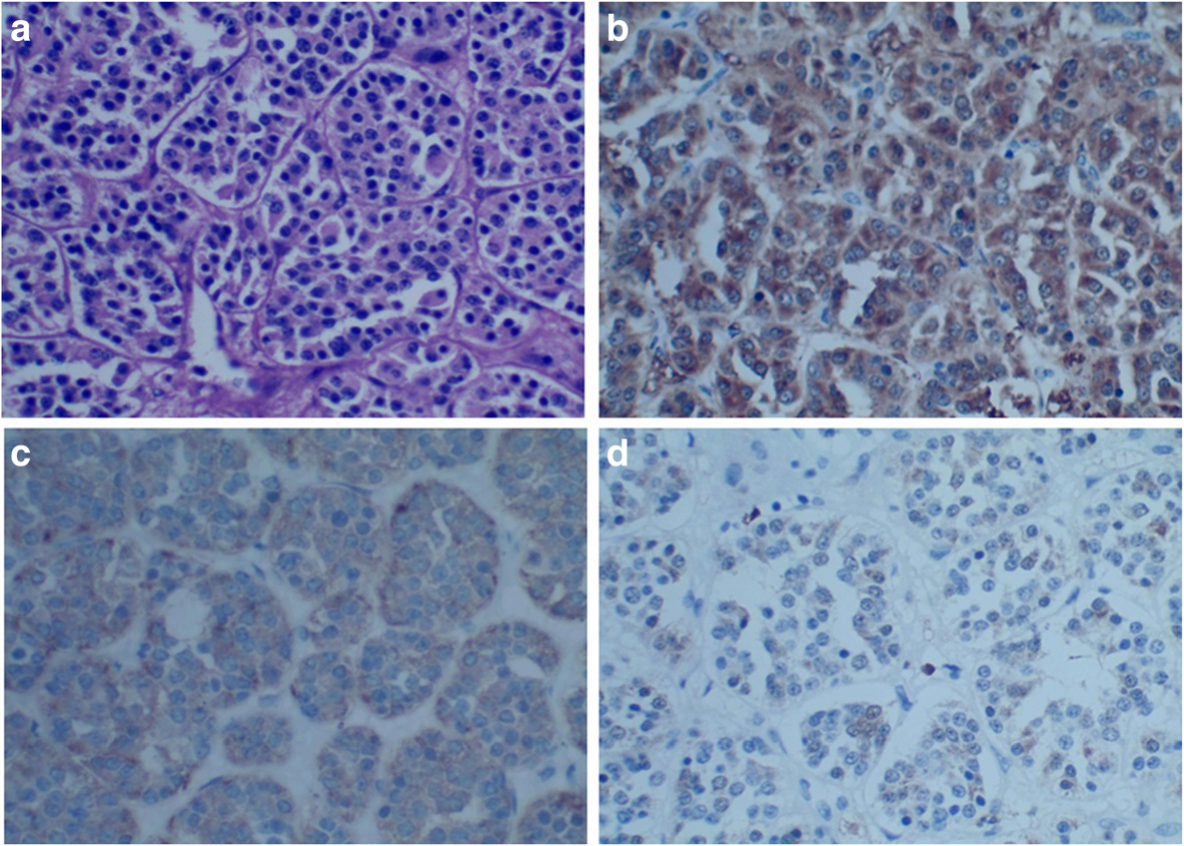
Pathology slides of the resected specimen with different stains. **a** Hematoxylin and eosin staining demonstrating the classic Zellballen pattern of paraganglioma (HE, ×400). **b** The chief cells showed positive staining to NSE (IHC, ×400). **c** The chief cells showed positive staining to Syn (IHC, ×400). **d** The sustentacular cells showed positive staining to S-100 (IHC, ×400)

A 42-year-old woman presented in September 2014 with recurrent upper abdominal pain for 3 months. She had no history of hypertension, headache, and palpitation. A physical examination revealed slight upper abdominal tenderness. Laboratory test results including liver function, renal function, and blood glucose were within normal ranges. Serum levels of CEA, CA19-9, and CA125 were normal. The level of 24-h urinary norepinephrine excretion was also normal. Unenhanced computed tomography (CT) revealed a 5.2 cm × 6.3 cm, solid, low density tumor on the body of the pancreas. On contrast-enhanced CT, the tumor demonstrated marked enhancement in the arterial phase (Fig. 1). Dilation of the pancreatic duct was noted at the tail of pancreas. No biliary dilation or liver lesions were detected. A diagnosis suspicion of a pancreatic neuroendocrine tumor was made before operation. The patient underwent middle segment pancreatectomy. The proximal pancreas was transected using a linear stapler and continuous suture using 4-0 prolene, the stump of the distal pancreas was anastomosis to the jejunum with duct-to-mucosa pancreaticojejunostomy. The two resection margins were frozen section to confirm tumor-free. During the operation, the patient’s blood pressure remained stable. Histological examination showed that the tumor was composed of well-defined nests of cuboidal cells separated by vascular fibrous septa, forming the classic Zellballen pattern (Fig. 2a). The chief cells showed positive staining to neuron-specific enolase (NSE) (Fig. 2b), chromogranin A (CgA), synaptophysin (Syn) (Fig. 2c), but showed negative response to vimentin (Vim), endomysial (EMA), cytokeratin (CK), insulin, and glucagon. The chief cells were surrounded by S-100 protein-positive sustentacular cells (Fig. 2d). The Ki67 labeling index was 1 % where no mitoses were observed. Region lymph nodes and the resected margins were free of tumor cells. Taking the morphological and immunohistochemical features into account, the diagnosis of primary pancreatic paraganglioma was confirmed. The patient’s postoperative course was uneventful and discharged on the 8th postoperative day. The patient received no subsequent adjuvant treatment, and had remained tumor free for 12 months after surgery.

## Discussion

Primary paraganglioma that arises in the pancreas is rare. Whether this tumor type is an extension of a retroperitoneal tumor of true visceral origin, derived from ectopic paraganglia, remains unknown. To the best of our knowledge, only 21 cases of pancreatic paraganglioma having been reported from 1943 to the present in the literature (Table 1) [1–18]. The mean age of the 21 cases reported in the literature was 57.6 years ranging from 19 to 85 years. Of those patients, 16 were women and five were men with the ratio of 3.2:1. The tumor was located in the head of the pancreas in 15 patients, 2 in the body, and 4 in the tail. The mean size of the tumors was 6.1 cm. Four cases were considered malignant, and six cases showed functional activity. Herein, we presented a 42-year-old woman with primary pancreatic paraganglioma, which located in the body of the pancreas. The patient had no symptoms of catecholamine excess, and the blood pressure remained stable during the operation, the norepinephrine levels was normal, therefore nonfunctional pancreatic paraganglioma was diagnosed.

**Table 1:** Twenty-one cases of pancreatic paraganglioma in the literature

Author	Year	Age (y)	Sex	Location	Size (cm)	Cystic or Solid	Function	Malignant	Treatment	Survival
Goodo [1]	1943	62	M	Body	1.5	Solid	No	No	–	Autopsy
Bartley [2]	1966	75	F	Tail	Goose egg	Cystic	Yes	No	DP	NM
Bartley [2]	1966	70	F	Head	Walnut	Cystic	Yes	No	TR	NM
Cope [3]	1974	72	F	Head	13	Cystic	No	No	TR	2Y(A)
Zamir [4]	1984	47	M	Body	10	Cystic	No	No	TR	6Y(A)
Fujino [5]	1998	61	M	Head	2.5	Solid	No	No	PD	5Y(A)
Parithivel [6]	2000	85	M	Head	6	Cystic	No	No	TR	3Y(A)
Ohkawara [7]	2005	72	F	Head	4	Cystic	No	No	TR	NM
Perrot [8]	2007	41	F	Tail	4.2	Solid	Yes	No	TR	18 M(A)
Tsukada [9]	2008	57	F	Head	2	Solid	No	No	TR	4Y(A)
Kim [10]	2008	57	F	Head	6.5	Solid	No	No	PPPD	NM
Paik [11]	2009	70	F	Tail	4.2	Solid	No	Yes	DP	NM
He [12]	2011	40	F	Head	4.5	Solid	No	No	NM	NM
Higa [13]	2012	65	F	Head	2	Solid	No	Yes	PD	10 M(A)
AI-Jiffry [14]	2013	19	F	Head	9.5	Solid	Yes	Yes	PD	3Y(A)
Zhang [15]	2014	50	F	Head	6	Solid	Yes	Yes	Laparotomy	4Y(D)
Zhang [15]	2014	63	M	Head	4	Solid	Yes	No	TR	3 M(A)
Borgohain [16]	2014	55	F	Tail	19	Solid	No	No	TR	10 M(A)
Straka [17]	2014	53	F	Head	8.5	Solid	No	No	PPPD	49 M(A)
Meng [18]	2015	54	F	Head	3	Solid	No	No	TR	NM
Meng [18]	2015	41	F	Head	6	Solid	No	No	TR	NM

*F* female, *M* male, *NM* not mentioned, *DP* diatal pancreatectomy, *PD* pancreaticoduodenectomy, *TR* tumor resection, *PPPD* pylorus preserving pancreaticoduodenectomy, *Y* years, *M* months, *A* alive, *D* dead

The location of pancreatic paragangliomas can usually be identified by abdominal ultrasonography, CT, or magnetic resonance imaging. In our current case, the pancreatic paraganglioma appeared as a solid mass on the body of the pancreas on CT scans, and marked enhancement was found on contrast-enhanced CT. The preoperative diagnosis of pancreatic paraganglioma is difficult, especially in nonfunctional cases. Functional cases are easier to diagnose because having symptoms of catecholamine excess such as hypertension, headache, and palpitation, and the urinary catecholamines are elevated. The confirmed diagnosis of paraganglioma mainly depends on histopathological and immunohistochemical findings as following (i) the classic Zellballen pattern composed of chief cells and sustentacular cells within the tumor; (ii) the chief cells showed positive staining to NSE, CgA, Syn, and negative for CK; and (iii) the sustentacular cells showed positive staining to S-100 or GFAP. Pancreatic paragangliomas are potential to be malignant [13–15], but factors predictive of malignant behaviors have not been well characterized. In general, malignant paragangliomas are defined as those that metastasize, recur, or show evidence of local invasion.

The first choice of treatment for primary pancreatic paraganglioma is complete surgical resection. Postoperative I^131^-metaiodobenzylaguanidine (I^131^-MIBG) radiotherapy has been advocated in cases proven to be malignant [14], chemotherapy and novel biologically targeted drugs could be the other reasonable choice. Although pancreaticoduodenectomy or pylorus preserving pancreaticoduodenectomy is recommended for paraganglioma of the pancreatic head, and distal pancreatectomy for tumors of the pancreatic body or tail, simple tumor enucleation also showed an equally good outcome. In the present case, middle segment pancreatectomy was performed being the tumor located in the body of the pancreas. Middle segment pancreatectomy procedure has the advantage of preserving normal pancreatic parenchyma to the most extent and consequently long-term endocrine and exocrine pancreatic function. The patient had an uneventful postoperative course. Being have no evidence of tumor invasion and metastases, the patient received no subsequent adjuvant treatment, and has been tumor free for 12 months after surgery. The long-term outcome is still in follow-up.

## Conclusions

We report a case of primary nonfunctional paraganglioma on the body of the pancreas. Middle segment pancreatectomy could be a reasonable procedure for such tumor. Pancreatic paraganglioma has malignant potential, and requiring close postoperative follow-up.

## Consent

Written informed consent was obtained from the patient for publication of this Case Report and any accompanying images. A copy of the written consent form is available for review by the Editor-in-Chief of this journal.

## Abbreviations

CgA: chromogranin A
CK: cytokeratin
CT: computed tomography
EMA: endomysial
MSP: middle segment pancreatectomy
NETs: neuroendocrine tumors
NSE: neuron-specific enolase
Syn: synaptophysin
Vim: vimentin
